# Cutaneous pseudolymphoma: a case series of three patients

**DOI:** 10.3389/fmed.2024.1507399

**Published:** 2025-01-17

**Authors:** Yue Yao, Yijia He, Zhuochen Wu, Jinfang Zhang, Guoqiang Zhang

**Affiliations:** ^1^Department of Dermatology, The First Hospital of Hebei Medical University, Shijiazhuang, Hebei Province, China; ^2^Subcenter of National Clinical Research Center for Skin and Immune Diseases, Shijiazhuang, Hebei Province, China; ^3^Hebei Technical Innovation Center for Dermatology and Medical Cosmetology Technology, Shijiazhuang, Hebei Province, China

**Keywords:** cutaneous pseudolymphoma, histopathological, immunohistochemical staining, thalidomide, leech bite

## Abstract

Cutaneous pseudolymphoma is a type of reactive lymphoid hyperplasia that pathologically and/or clinically mimics cutaneous lymphoma. It has a variety of pathogenic factors. However, in many cases, no etiology can be identified. The histopathological findings of cutaneous pseudolymphoma can only be used as the basis for suspected diagnosis. Clinical data, further diagnostic tests and follow-up are needed to confirm the diagnosis. This article reports three cases of cutaneous pseudolymphoma, with different etiology, clinical manifestations and treatment methods. The purpose of this article is to emphasize that the etiology and clinical manifestations of cutaneous pseudolymphoma are diverse, and targeted treatment strategies and long-term follow-up are needed.

## Introduction

Cutaneous pseudolymphoma (CPL) is a reactive polyclonal lymphoproliferative disorder associated with known and unknown etiologies. It may be localized or scattered in the skin and may be transient or persist for several years. Although topical and local injections of glucocorticoids are the most common treatments for CPL, there is no definitive treatment for cases that are refractory to these treatments. In some cases, the rash resolves spontaneously (1). Here we report three cases of CPL with different etiology, clinical manifestations and treatment methods, aiming to emphasize that the etiology and clinical manifestations of cutaneous pseudolymphoma are diverse. Individualized treatment for different clinical data and long-term follow-up are needed.

## Case presentation

### Case 1

**Figure 1:**
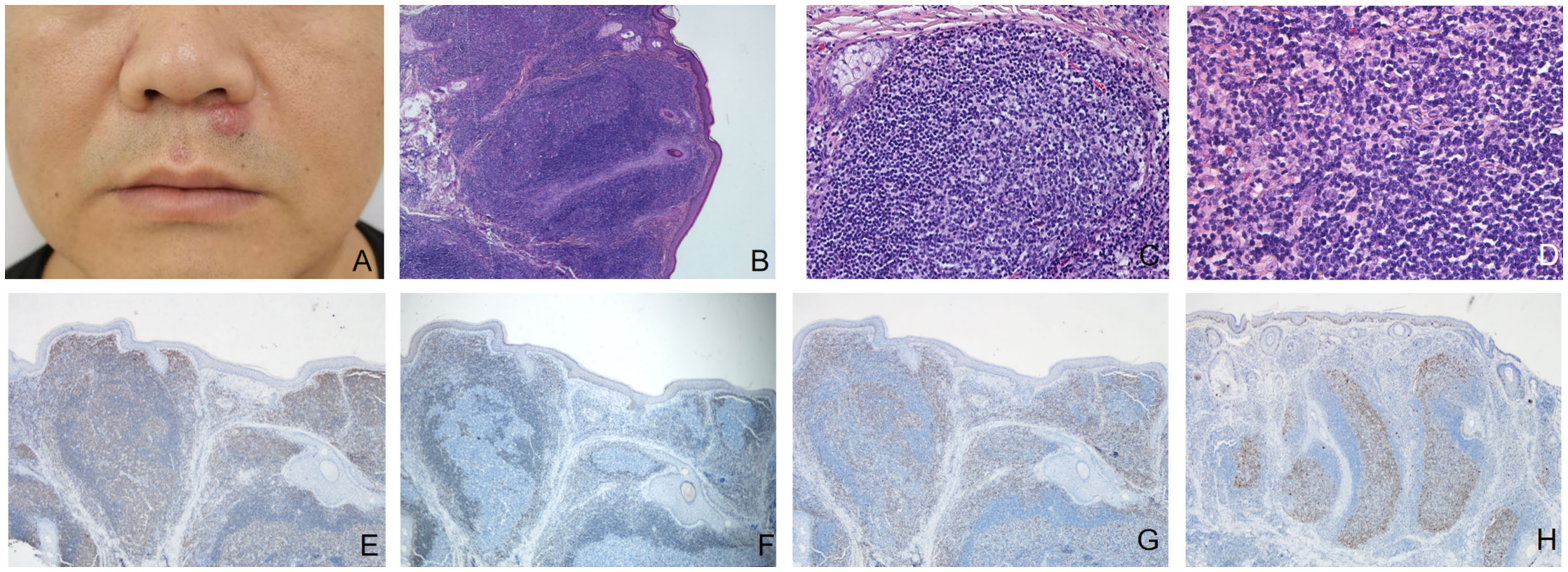
**(A)** A firm reddish nodule 6 mm in diameter under the nose. **(B)** The epidermis was almost normal, the basal cells were intact, and a large number of cell clusters with large and dark nuclei massively infiltrated the dermis and subcutis, and the adnexa in the clusters was reduced (hematoxylin-eosin, original magnification ×40). **(C)** Lymph follicle with well-defined germinal center and mantle zone (hematoxylin-eosin, original magnification ×200). **(D)** Dense lymphoid infiltrates along with a minority of eosinophils were seen in the dermis (hematoxylin-eosin, original magnification ×400). **(E)** Immunohistochemical staining (original magnification ×40) showed CD20 was predominantly positive in the infiltrating cells. **(F)** The cells in the center of nodular infiltration were negative for Bcl-2. **(G)** CD3-positive cells were mainly scattered around the center of nodular infiltration. **(H)** In Ki-67 stain, the proliferative activity is elevated and mainly confined to the center of cells with nodular infiltration.

A man in his 40s presented with a 6-month history of one firm reddish nodule under his nose (Figure 1A) without symptom. There was no palpable enlargement of the superficial lymph nodes. The patient had a medical history of hyperlipidemia and gout. The patient stated that he had not been exposed to any arthropods, including ticks, and had no history of allergy. He had been treated with fire needle and laser therapy, but the effect was poor. Complete blood cell count was normal. Histopathologic examination showed nodular infiltration of a large number of dense lymphoid cells and some plasma cells in the dermis and subcutaneous tissue with no obvious nuclear atypia, mixed with few eosinophils and histiocytes, and the number of appendages within the nodules was decreased (Figures 1B–D). Immunohistochemical staining showed CD20 (Figure 1E) was predominantly positive in the infiltrating cells. The cells in the center of nodular infiltration were positive for Bcl-6 and negative for Bcl-2 (Figure 1F). CD3 (Figure 1G) and CD4-positive cells were scattered around the center of nodular infiltration. In Ki-67 stain, the proliferative activity was elevated and mainly confined to the center of cells with nodular infiltration (Figure 1H). A diagnosis of cutaneous pseudolymphoma (CPL) was made. The patient was treated with surgical resection, showing a favorable outcome and no recurrence after 2 years follow-up.

### Case 2

**Figure 2:**
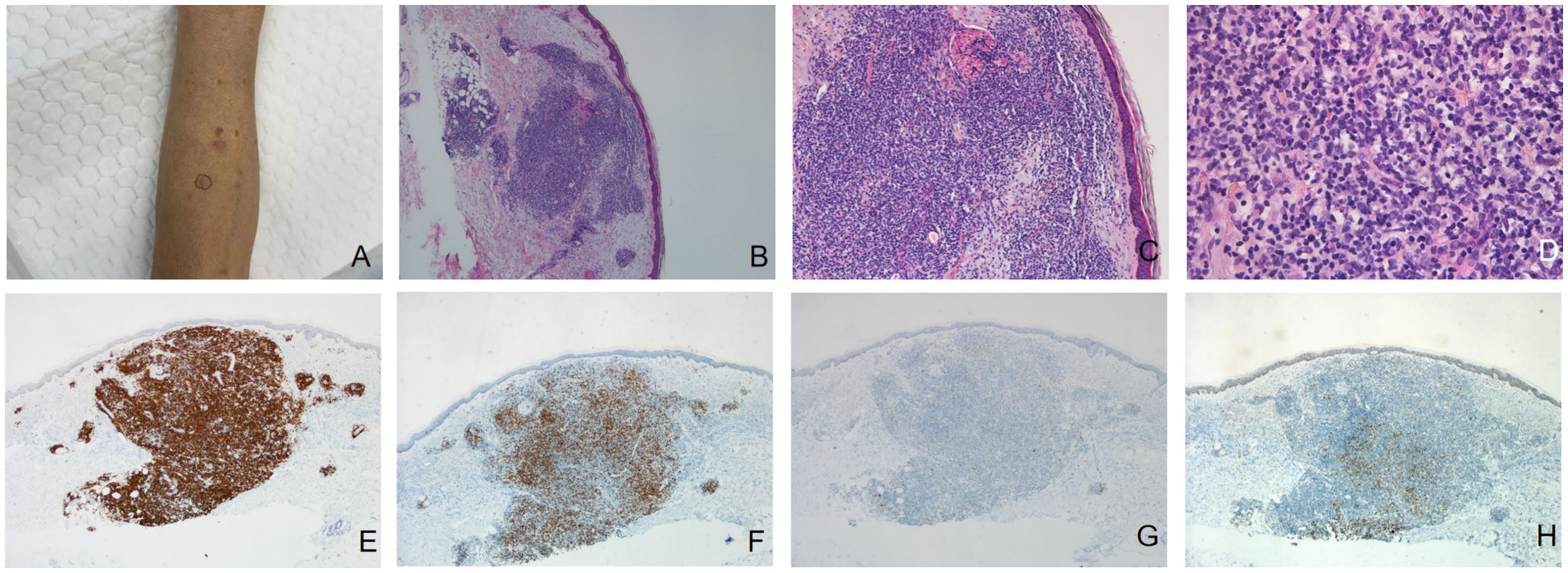
**(A)** Multiple red hard and smooth papules and nodules with a diameter of 0.5–2 cm on the left upper limb. Dense, nodular lymphocytic infiltrate in the dermis [hematoxylin-eosin, original magnification: **(B)** ×40; **(C)** ×100]. **(D)** A dense infiltration of lymphocytes, histiocytes, and plasma cells was observed in the dermis (hematoxylin-eosin, original magnification ×400). **(E)** Immunohistochemical staining results (original magnification ×40) revealed that most of the infiltrating cells were positive for the B-cell makers CD20. **(F)** Some lymphoid cells was positive for the T-cell maker CD3. Bcl-2 **(G)** was negative and Bcl-6 **(H)** was weakly positive in the center of the nodular infiltrated cells in the dermis.

A 69-year-old woman presented for the evaluation of multiple red firm and smooth papules and nodules with a diameter of 0.5–2 cm on both upper limbs (Figure 2A) and the scalp, which had occurred without obvious causes 6 months ago. She had a previous history of bone tuberculosis, which was cured by surgery. The patient had no history of arthropod exposure. Routine complete blood count showed an increase in neutrophils. Chest computed tomography revealed a slight thickening of the bronchial walls in both lungs, with surrounding patchy high-density shadows, suggesting infectious lung diseases, and a few calcifications in the upper lobes of both lungs, most possibly caused by old tuberculosis. Histopathological analysis of the papule on the left upper limb showed chronic inflammation of the skin tissue with atypical hyperplasia of local dermal lymphocytes (Figures 2B–D). Immunohistochemical staining results revealed that most of the infiltrating cells were positive for the B-cell makers CD20 (Figure 2E) and CD79a, and some lymphoid cells were positive for the T-cell maker CD3 (Figure 2F). Bcl-2 (Figure 2G) was negative and Bcl-6 (Figure 2H) was weakly positive in the center of the nodular infiltrated cells in the dermis. The patient was preliminarily diagnosed with CPL. Considering that the lesions were relatively limited with no obvious subjective symptoms, further observation has been recommended. After a 8-month follow-up, the patient reported that the original rash had resolved, with occasional new rashes.

### Case 3

**Figure 3:**
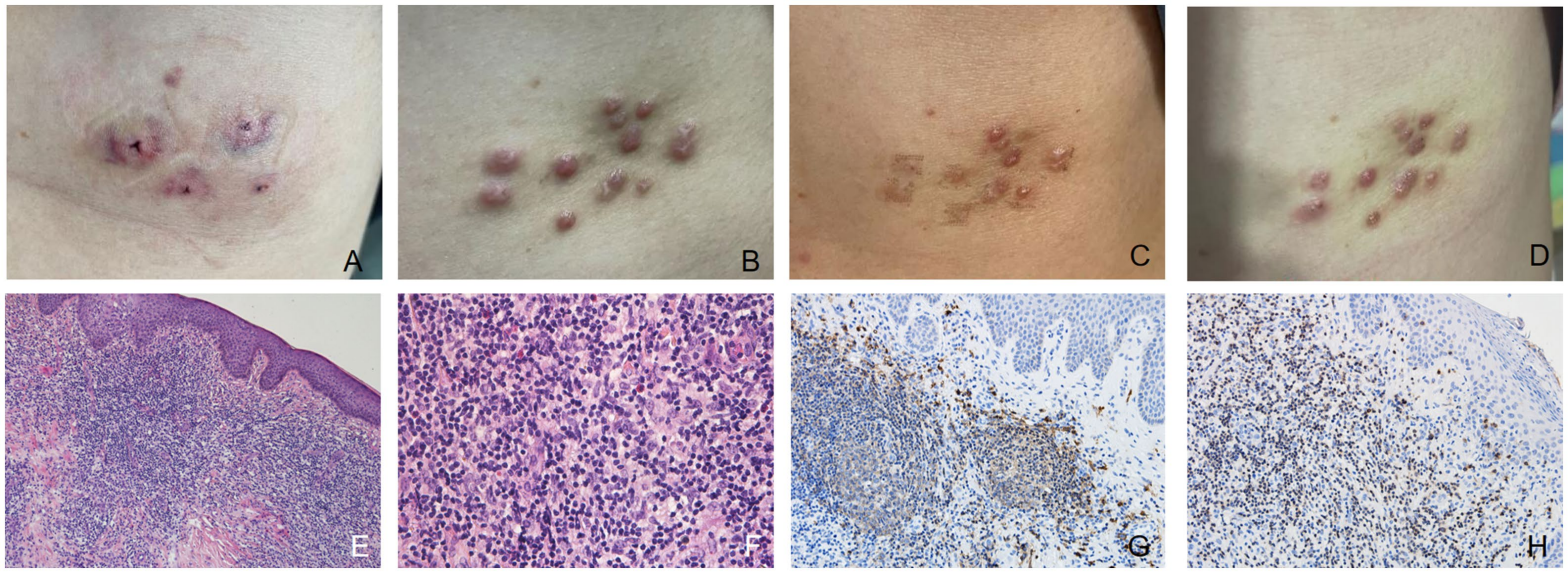
**(A)** After the first hirudotherapy, red patches appeared with a “Y” shaped depression visible in the center and a purplish halo surrounding it. **(B)** Multiple firm and red nodules developed at the site where the leeches were applied. **(C)** After being treated with intralesional glucocorticoids and fractional laser, some lesions thinned or even nearly resolved. **(D)** The rashes that had thinned or even disappeared before reappeared a few weeks later. **(E,F)** The epidermis showed irregular hyperplasia, with intact basal cells, and infiltration of a large number of lymphocytes, histiocytes, plasma cells and a small number of eosinophils in the dermis and subcutis, without obvious atypia (hematoxylin-eosin, original magnification: **E** ×100; **F** ×400). **(G)** Immunohistochemical stains (original magnification ×200) showed that the expression of B cell markers CD20 was positive. **(H)** The expression of T cell markers CD3 was positive.

A 25-year-old woman presented with scattered reddish nodules on her left chest. She had undergone hirudotherapy for a 5 × 6 cm mass on her left chest in a private clinic (Figure 3A). A few days later the nodules developed at the site where the leeches were applied without symptom (Figure 3B). Physical examination revealed multiple firm and red nodules. The patient had been previously in good health. She denied any prior arthropod exposure and allergy in her medical history. Complete blood cell count was normal. Syphilis and HIV tests were negative. Histopathological biopsy showed irregular epidermal hyperplasia, with abundant infiltration of lymphocytes, histiocytes, plasma cells and few eosinophils in dermis and subcutaneous areas, and no obvious atypia (Figures 3E,F). Immunohistochemical stains showed that the expression of B cell markers CD20 (Figure 3G) and CD79a and T cell markers CD3 (Figure 3H) and CD4 were positive, while the expression of Bcl-2 was negative. The proliferative rate, highlighted with Ki-67, was elevated in dermal infiltrate. A favored diagnosis of CPL secondary to the leeches was made. Intralesional glucocorticoids and fractional laser were applied, after which the lesions thinned or even nearly resolved (Figure 3C), but they recurred several weeks later (Figure 3D). After confirming the absence of pregnancy and abnormal nerve conduction, the patient’s treatment regimen was subsequently adjusted to oral thalidomide 25 mg b.i.d. combined with topical potent steroid. This patient was instructed to use contraception strictly during the medication period and 3–6 months after drug withdrawal. Most of the lesions were resolved after 1.5 months. No adverse effects were observed. She is currently undergoing further follow-up.

## Discussion

CPL is a kind of lymphocytic proliferation with benign biological behavior that resembles cutaneous lymphoma histopathologically or clinically. Various causative factors have been reported, including medications, bacterial spirochetes such as *Borrelia burgdorferi*, tattoo dyes, arthropod bites, etc. However, the etiology cannot be determined in many cases, which results in idiopathic CPL (2). CPL is classically divided into T-cell CPL, B-cell CPL and mixed T/B-cell CPL according to histopathological patterns (1, 3). Immunohistochemistry distinguishes lymphocytes based on their immunophenotype (surface markers), such as assignment to B or T cells. Mixed infiltration of B and T cells is more likely to be CPL (3). The infiltration of nodular B-cell CPL is predominantly composed of B-cells expressing typical B-cell markers, such as CD19, CD20, CD79a and PAX-5. Reactive germinal centers express bcl-6 and are negative for bcl-2. The proliferation index Ki-67 or Mib-1 is elevated, especially in the germinal centers (4). Mitteldorf et al. (4) proposed categorizing CPL into four major groups based on histopathological and clinical data: nodular pseudolymphomas, pseudolymphomas as simulators of mycosis fungoides, other pseudolymphomas and intravascular pseudolymphomas. All three cases in our report are nodular pseudolymphomas. CPL often occurs on the face, limbs, around the breast and other areas, with single or multiple reddish nodules or plaques. The lesions are usually asymptomatic, and symptoms such as burning and pruritus can also be observed (5).

CPL may be histopathologically indistinguishable from cutaneous lymphoma. Several histopathological characteristic favour lymphoma over CPL: ① deep infiltrate down to the subcutaneous fat; ② monomorphic infiltrate; ③ high-grade atypical lymphocytes (changes in nuclear morphology, nuclear density and nucleus/cytoplasm ratio). B-cell markers (e.g., CD79a^+^, CD20^+^, Pax-5^+^) are positive in the infiltrating cells in primary cutaneous marginal zone lymphoma (PCMZL), with Bcl-2 being positive, and Bcl-6 negative. Immunoglobulin light chain restriction for kappa or lambda (ratio of at least 5:1 or 10:1) was observed in 85% of PCMZL cases. Due to limited laboratory and financial capabilities, this test was not conducted on three of our patients. 60–70% of PCMZL has been observed clonal B cells, while some PCMZLs showed no monoclonal B cells. However, light chain restriction could also be seen in CPLs (4). Progression of CPL to cutaneous lymphoma has been reported in only a handful of cases, so patients must be followed up regularly (6). CPL may also be confused with polymorphic light eruption, cutaneous sarcoidosis, lupus erythematosus and histiocytosis (7). A comprehensive consideration of clinical, pathological, immunophenotypic and molecular features as well as long follow-up outcomes are required for the differential diagnosis.

Due to rarity and complexity of CPL, treatment recommendations are based mainly on case reports, small case series and expert opinions, and CPL may undergo spontaneous regression. The primary step in the treatment of CPL is to prevent re-exposure to the inducing agents, such as drugs, bacterial spirochetes, tattoo dyes, arthropod bites, *Hirudo medicinalis*, etc. Topical, intralesional and systemic corticosteroids, cryotherapy, radiation therapy, laser therapy and oral antimalarials, tetracycline and thalidomide can be used to treat CPL, whereas solitary CPL lesions can be removed by surgery (4). However, the efficacy is limited and relapse is frequent. The strategy for therapy depends on factors such as the patient’s age, comfort level, expectations and the location of the lesion. In our report, the patient in case 1 had one isolated skin lesions on his face that were surgically resected with cosmetic suturing. In case 2, the rash resolved spontaneously with occasional new rash. It is recommended that patients return to the clinic in time if the rash increases and becomes larger in a short time. For CPL caused by leech therapy, intralesional corticosteroid injections combined with fractional laser treatment were initially used but resulted in lesion recurrence after a few weeks, which may be related to the residual hirudin in the body. Compared to the previously published cases of leech-induced CPL (5, 7–15) (Table 1), the treatment in our case was more intractable. The application of glucocorticoids does not eliminate the skin lesions. After the inadequate effect of standard drug therapy, surgical resection was not appropriate considering the extensive and scattered lesions, and the patient was worried about postoperative scarring. Thalidomide was another treatment option for refractory CPL (16). After confirming the absence of pregnancy and abnormal nerve conduction, the patient was then switched to oral 25 mg b.i.d. thalidomide along with topical potent steroids. Considering that the patient was a female in the reproductive age and that thalidomide had a teratogenic effect, it is advised that the patient strictly use contraception during medication and for 3–6 months after stopping medication, and take medication regularly. Most of the lesions had been resolved after 1.5 months. No adverse effects were observed. The patient is currently being followed up. As the application of leech therapy in various medical fields increases, adverse skin reactions are becoming more frequent. Therefore, this rare cause should also be considered in the diagnosis and differential diagnosis of pseudolymphoma.

**Table 1:** Literature review of cutaneous pseudolymphoma caused by leeches.

Article	Age/gender	Site	Reason for applying leeches	Manifestations of lesions	Symptom	Leech-applying person	Prominent cell type	Treatment	Outcome
Yao et al. (case 3)	25/F	Left chest	A 5 × 6 cm mass	Nodules	Asymptomatic	Private practice	Mixed	IL CS, laser, thalidomide, topical CS	Most of lesions resolved after 1.5 months
Mittal et al. (8)	20/M	Leg	An accidental bite	Nodule	Pruritus	An accidental bite	N/A	IL CS	Complete resolution, no recurrence for 1 year
Sepaskhah et al. (5)	44/F	Leg	Erythema nodosum	Papules	Pruritus	General practitioner	N/A	IL, topical, oral CS	Resolved after 1.5 months, no recurrence for 6 months
Sadati et al. (9)	45/F	Leg	Varicosities	Papules	Pruritus	General practitioner	N/A	Topical CS, cryotherapy	Resolved after 3–4 weeks
Temiz et al. (7)	54/M	Neck	N/A	Plaques	Pruritus	N/A	T cell	IL CS	Resolved after 1 month, no recurrence for 6 months
Aktaş et al. 2018 (10)	65/F	Lower back	Lumbar pain	Nodules	Pruritus	N/A	N/A	IL CS, cryotherapy	No subjective symptoms after 3 weeks
Tupikowska et al. (11)	38/F	Pubis	Uterine myoma	Nodules	Pruritus	Friend	Mixed	IL, topical, IM CS, cryotherapy	A slow regression of skin lesions after 20 weeks
Altamura et al. (12)	50/F	Back	Fibromyalgia	Nodules papules	Pruritus	N/A	B cell	Topical CS	Resolved after 3–4 weeks, no recurrence for 15 months
Khelifa et al. (13)	77/F	Lower back	Low back pain	Nodules	Asymptomatic	Private, alternative medicine clinic	Mixed	Topical CS, IL CS	Favorable
Choi and Kim 2012 (14)	52/M	Lower eyelids	Dark circles	Nodules	N/A	Non-medical personnel	T cell	IL CS	Mostly cleared up for 3 months
Smolle et al. (15)	56/F	Lower leg	Venous insufficiency	Nodules scar	N/A	N/A	B cell	IL CS	Gradual clearing of the skin lesions

F, female; M, male; N/A, not available; IL, intralesional; CS, corticosteroid; IM, intramuscular.

Approximately 1% of *Borrelia burgdorferi* sp. infections manifest as B-cell CPL, often presenting as an isolated red to purple nodule in the earlobes, nipples, scrotum, trunk or extremities. The diagnosis can be confirmed based on histopathology and detection of *Borrelia burgdorferi* sp. DNA by PCR. Clinical data such as tick bites, serology and Warthin–Starry silver staining are also helpful for diagnosis (1). Tingible body macrophages are seen in all cases of *Borrelia*-associated nodular CPL, which is a helpful clue (4, 17). Although all three patients denied a history of ticks or other arthropod bites, and they did not live in an area with a high incidence of Lyme disease, B-cell CPL caused by *Borrelia burgdorferi* sp. cannot be considered without caution, and the inability to perform any examination for *Borrelia burgdorferi* sp. is the major limitation of this study due to the limited laboratory capabilities and the patients’ financial capacity. In the future, we will further improve the diagnosis and treatment of CPL and conduct regular follow-up of these three patients.

## Conclusion

We reported three cases of CPL with different clinical manifestations and provided with different therapy. Although long-term regular follow-up is still needed, this report aims to remind us that the etiology and clinical manifestations of CPL vary, and different appropriate treatment strategies need to be adopted.

## Data Availability

The raw data supporting the conclusions of this article will be made available by the authors, without undue reservation.
